# Measuring and mapping the global burden of antimicrobial resistance

**DOI:** 10.1186/s12916-018-1073-z

**Published:** 2018-06-04

**Authors:** Simon I. Hay, Puja C. Rao, Christiane Dolecek, Nicholas P. J. Day, Andy Stergachis, Alan D. Lopez, Christopher J. L. Murray

**Affiliations:** 1 Institute for Health Metrics and Evaluation, University of Washington, 2301 5th Ave, Seattle, WA 98121 USA; 2 Big Data Institute, University of Oxford, Li Ka Shing Centre for Health Information and Discovery, Old Road Campus, Oxford, OX3 7LF UK; 3 Centre for Tropical Medicine and Global Health, Nuffield Department of Medicine, University of Oxford, Old Road Campus, Roosevelt Drive, Oxford, OX3 7FZ UK; 4 Mahidol–Oxford Tropical Medicine Research Unit, Faculty of Tropical Medicine, Mahidol University, 420/6 Rajvithi Road, Bangkok, 10400 Thailand; 5 School of Pharmacy, University of Washington, H-362B Box 357631, Seattle, WA 98195 USA; 6 Melbourne School of Population and Global Health, The University of Melbourne, Level 5, 207 Bouverie St, Carlton, VIC 3053 Australia

**Keywords:** Antimicrobial resistance, Drug-resistant infections, Microbial, Anti-infective agents, Antimicrobial drugs, Global health, Global burden of disease, Public health

## Abstract

The increasing number and global distribution of pathogens resistant to antimicrobial drugs is potentially one of the greatest threats to global health, leading to health crises arising from infections that were once easy to treat. Infections resistant to antimicrobial treatment frequently result in longer hospital stays, higher medical costs, and increased mortality. Despite the long-standing recognition of antimicrobial resistance (AMR) across many settings, there is surprisingly poor information about its geographical distribution over time and trends in its population prevalence and incidence. This makes reliable assessments of the health burden attributable to AMR difficult, weakening the evidence base to drive forward research and policy agendas to combat AMR. The inclusion of mortality and morbidity data related to drug-resistant infections into the annual Global Burden of Disease Study should help fill this policy void.

## Background

Antimicrobial resistance (AMR) occurs when bacteria, viruses, fungi, and parasites adapt to antimicrobial drugs, resulting in drug inefficiency and persistent infections, with a subsequent increase in the risks of severe disease and transmission. AMR is a major global threat to the health of populations, endangering the ability to prevent and cure a wide range of infectious diseases [1–3]. While AMR occurs naturally, the emergence and spread of new resistance mechanisms may have been greatly accelerated by the overuse and misuse of antimicrobials [4]. In many countries, antibiotics are given without professional oversight and are inappropriately used in both people and animals; important examples of such misuse include the consumption of antibiotics by people with common viral infections or when given to farm-raised fish or livestock as growth promoters [4–7]. Microbes that are resistant to antimicrobials are found in people, animals, food, and the environment, and can spread between humans and animals, and from person to person [8]. Further, poor infection control, inadequate sanitary conditions, and inappropriate food handling may facilitate the spread of AMR within populations [9].

There are many challenges to estimating the burden of AMR. Primarily, there is limited and unreliable current and historical information on the geographical distribution, prevalence, and incidence of AMR and its health burden, making the burden of AMR difficult to measure and limiting our ability to devise geographically explicit strategies for its control [10, 11]. Disparate data sources from public and private sectors are often not collated at the national and international levels and contain little information on individual patients and their outcomes. Furthermore, there are fundamental issues of selection bias in terms of who is tested for AMR and whether or not that information is entered into facility-based laboratory data systems. Additionally, systematic efforts are yet to be made to quantify antimicrobial drug utilization patterns, which would yield important data to address AMR. Protocols for diagnostic methods and data collection need to be standardized to allow an accurate depiction of the true health burden of AMR to be constructed. These problems are exacerbated in low- and middle-income countries, where there is often inadequate surveillance, minimal laboratory capacity, and limited access to essential antimicrobials.

As the threat of AMR continues to grow, more work is needed to supplement current surveillance and methodologically inconsistent research regarding the global epidemiology and impact of AMR. Some studies have addressed the challenges to measuring the burden of AMR and also provide estimates of prevalence of resistance for particular pathogen-antimicrobial drug combinations in various locations [1, 3, 12]. However, major gaps in data on prevalence and incidence as well as on types of resistance, treatment failures, and studies on the attributable mortality and morbidity of AMR, particularly in low- and middle-income countries, have made it nearly impossible to reliably estimate the global impact of AMR. To combat AMR, policymakers and other stakeholders have issued calls to action, including a focus on broad improvements to surveillance of the current global resistance situation, support for and prioritization of new diagnostics, antimicrobials and vaccines, and improved stewardship of existing antimicrobials to avoid further selection and emergence of resistant bacteria [1, 2, 12, 13]. As a critical input to these actions, a high-quality evidence base is needed to support surveillance and data collection efforts, as well as to inform global policy priorities, set clear international standards and guidelines, establish intervention priorities, and support investment decisions.

## Quantifying the global burden of AMR

Here, we present a new project (“AMR project”) to provide rigorous quantitative evidence of the burden of AMR, to increase awareness of AMR, to support better surveillance of AMR, and to foster the rational use of antimicrobials around the world. An important aspect of this AMR project is the integration of AMR burden with the larger Global Burden of Diseases, Injuries, and Risk Factors (GBD) Study at the Institute for Health Metrics and Evaluation.

The GBD is an ongoing comprehensive global research program that provides comparable estimates of mortality and disability resulting from 328 disease and injury causes, as well as from 84 risk factors, across age and sex groups, over time and space [14–17]. This AMR project will take advantage of the established infrastructure of the GBD, which involves over 3200 collaborators in 140 countries and three non-sovereign locations around the world, and will be conducted under a strategic partnership with the Big Data Institute and the Centre for Tropical Medicine and Global Health at the University of Oxford, UK. The GBD has the largest known existing repository of epidemiological data, which is used to compare the loss of healthy life due to a particular health disparity, such as AMR, around the world relative to other causes of disability and mortality. As with other research areas in the GBD, this research will undergo a high level of scientific scrutiny by leveraging feedback on data, modelling, and results from existing partnerships and collaborations. Perhaps more importantly, including AMR in the GBD will ensure that the resulting estimates comply with the rigorous, evidence-based framework that characterizes the GBD effort. Annual updates to data and results enable ongoing, improved annual assessment of the AMR burden.

The strategy we will pursue in estimating the burden of AMR has several dimensions. First, because of the important role of sepsis involving a drug-resistant organism as an intermediate pathway to death from AMR, we will analyze the burden of all forms of sepsis in the GBD. Second, we will collate and analyze the data gathered by the public and private sectors on resistant bacteria present in various human samples (blood, urine, stool, wounds, etc.). Third, we will systematically review published and unpublished sources on the relative case-fatality rate for drug-resistant versus drug-sensitive infections for different clinical syndromes. These components will allow estimation of the burden of resistance for different drug-organism combinations. Over time, we will systematically expand the number of combinations included in the GBD assessment. The initial objective of the burden of disease assessment for AMR will include a comprehensive synthesis of existing global AMR data on selected bacterial pathogens (listed in Table 1), developing analytical methods to estimate the fraction of burden from causes is attributable to AMR, and producing disease burden estimates and incorporating them into interactive visualizations and maps for public access.

**Table 1:** Geospatial maps of the prevalence and incidence of resistance of selected bacteria-antibacterial drug combinations will be created

Bacteria	Antibacterial drug(s)
*Escherichia coli*	Third-generation cephalosporins, fluoroquinolones
*Shigella* species	Fluoroquinolones
*Klebsiella pneumoniae*	Third-generation cephalosporins, carbapenems
*Streptococcus pneumoniae*	Penicillin
*Staphylococcus aureus*	Methicillin
*Salmonella* Typhi and Paratyphi	Fluoroquinolones, chloramphenicol
Non-typhoidal *Salmonellae*	Fluoroquinolones
*Neisseria gonorrhoeae*	Third-generation cephalosporins
*Mycobacterium tuberculosis*	First-line – isoniazid, rifampicin Second-line – fluoroquinolones, amikacin, capreomycin, kanamycin

## Conclusions

Results from this effort will contribute to the assessment of the burden of AMR over time, allowing an evaluation of current and past magnitude and geographical dispersion of the hazard, thus providing essential health intelligence to guide interventions and policies, as well as a benchmark for measuring the impact of interventions on future burden. By incorporating this work into the GBD, it will enable comparison of the loss of healthy life due to AMR for populations around the world relative to other causes of disability and mortality by age, sex, and location over time. In addition, updates to data and results will enable ongoing annual assessment of the incidence of AMR to track progress towards reducing its burden. This aim will benefit from and be supported by similar streams of work within this AMR project that will map the prevalence and incidence of resistance of selected bacteria-antibacterial drug combinations at the highest geographic resolution possible (Table 1). Analyses such as these are urgently required to provide accurate and timely data on the magnitude of and trends in AMR burden across the world. Accurate assessments of AMR burden can be used to inform treatment guidelines and agendas for decision-making, surveillance and research, to detect emerging problems, help guide investments in combatting AMR, and monitor trends to inform global strategies, as well as to facilitate the assessment of interventions over time.
